# Three-dimensional measurement of LV and RV dimensions using prospective self-gating for simultaneous compensation of cardiac and respiratory motion

**DOI:** 10.1186/1532-429X-11-S1-O102

**Published:** 2009-01-28

**Authors:** Robert Manka, Peter Boesiger, Martin Buehrer, Sebastian Kozerke

**Affiliations:** 1 German Heart Institute Berlin, Berlin, Germany; 2 Institute for Biomedical Engineering, Zürich, Switzerland

**Keywords:** Right Ventricular, Left Ventricular Mass, Image Quality Score, Lower Image Contrast, Cine Sequence

## Purpose

To compare three-dimensional (3D) balanced steady-state free precession (SSFP), prospective self-gating technique [1] without ECG triggering and breath-holding for the assessment of left ventricular (LV) and right ventricular (RV) function in the heart in comparison to standard 2D, multiple breath-hold SSFP cine imaging.

## Methods

Data were acquired in 15 subjects (10 volunteers, 5 patients) using a 1.5 T system with a five element cardiac array coil. In each subject a standard multi-slice, multi-breathhold 2D cine SSFP sequence was performed with complete ventricular coverage. Additionally, a three-dimensional cine sequence with prospective self-gating [1] with complete ventricular coverage was acquired during free breathing. LV and RV end-systolic volume (ESV) and end-diastolic volume (EDV) and LV mass were calculated for each method. With both imaging techniques, a patient-based analysis of image quality was performed with grading on a four-point scale, referring to the visibility of the endocardial border (excellent (4), good (3), moderate (2) and nondiagnostic (1)).

## Results

**Figure 1:**
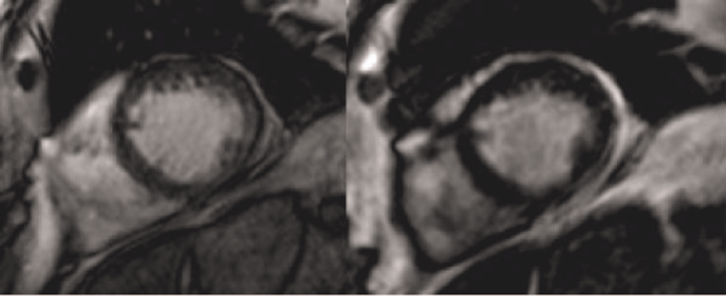
**End-diastolic (ED) frame of the left and right ventricle (mid-ventricular slice in short-axis orientation); left: multi-breath-hold, standard SSFP, right: free breathing prospective self-gating**.

Good agreement between LVEDV, LVESV, LV mass, LVEF, RVEDV, RVESV, and RVEF calculated for the standard 2D and the 3D prospective self-gating method (concordance coefficients 0.99, 0.99, 0.99, 0.90, 0.95, 0.95 and 0.91, respectively). The mean bias (95% confidence interval (CI) for each parameter was; LVEDV: -0.3% (-5.2 to 4.6), LVESV: 0.3% (-5.4 to 6.0), LV mass: -0.8% (-8.3 to 6.8), LVEF: -0.2% (-2.7 to 2.4), RVEDV: 4.5% (-9.6 to 18.6), RVESV: 3.8% (-11.2 to 18.7), RVEF: 1.0% (-4.7 to 6.7). The overall image quality score for prospective self-gating (2.7 ± 0.8) was lower when compared to standard SSFP (3.9 ± 0.4; p < 0.01). Figure 1 shows representative images from one patient.

## Discussion

Three-dimensional, free-breathing, prospective self-gating MRI enabled accurate assessment of LV and RV quantitative parameters when compared to standard multi-slice, multi-breathhold SSFP cine imaging. Image quality with prospective self-gating was rated lower relative to the reference ECG triggered, multiple breathhold scans due to lower image contrast between blood and myocardium and residual motion artefacts.
